# Efficacy of a high-dose proton pump inhibitor in patients with gastroesophageal reflux disease: a single center, randomized, open-label trial

**DOI:** 10.1186/s12876-020-01410-z

**Published:** 2020-08-18

**Authors:** Jae Ho Cho, Cheol Min Shin, Hyuk Yoon, Young Soo Park, Nayoung Kim, Dong Ho Lee

**Affiliations:** Department of Internal Medicine, Seoul National University Bundang Hospital, 82, Gumi-ro 173 Beon-gil, Bundang-gu, Seongnam, Gyeonggi-do 13620 South Korea

**Keywords:** Rabeprazole, Proton pump inhibitor, Gastroesophageal reflux disease, Extraesophageal manifestation

## Abstract

**Background:**

The extraesophageal manifestations of gastroesophageal reflux disease (GERD) are more difficult to manage than the typical symptoms. The efficacy of high-dose and standard-dose proton pump inhibitors against these atypical symptoms is not yet established.

**Methods:**

In this single center, randomized, open-label study, patients with GERD received rabeprazole for 8 weeks, either 20 mg once daily (standard-dose group) or 20 mg twice daily (high-dose group). Patients were assessed before treatment and at weeks 4 and 8 with a 5-graded scale questionnaire consisting of 2 typical symptoms (heartburn and acid regurgitation) and 8 atypical symptoms (chest pain, cough, globus, wheezing, laryngopharyngitis, hoarseness, belching, and dysphagia). Sufficient improvement of reflux symptoms was defined as ≥50% reduction from the initial questionnaire score.

**Results:**

Final analyses included 35 patients in the standard-dose group and 38 patients in the high-dose group. The rate of sufficient improvement for typical symptoms was significantly higher in the high-dose group than in the standard-dose group (100.0% vs. 84.0%, *P* = 0.040). For atypical symptoms, the rate of sufficient improvement tended to be higher in the high-dose group than in the standard-dose group (82.4% vs. 63.0%, *P* = 0.087). Scores of typical and some atypical symptoms (cough and globus) improved after treatment, with significant inter-group differences in time-course changes.

**Conclusions:**

High-dose rabeprazole is more effective for relieving typical GERD symptoms and some atypical symptoms such as cough and globus than a standard-dose regimen.

**Trial registration:**

This research was enrolled in a registry of clinical trials run by United States National Library of Medicine at the National Institutes of Health (ClinicalTrials.gov Protocol Registration and Results system ID: NCT04001400). This study was registered on June 26, 2019 - Retrospectively registered.

## Background

Gastroesophageal reflux disease (GERD) is a condition in which reflux of stomach contents into the esophagus causes troublesome symptoms and complications [1]. The prevalence of GERD varies from approximately 2.5 to 40% worldwide, and is higher in Western countries and lower in East Asia [2–5]. Typical symptoms of GERD are heartburn and regurgitation, but this disease also presents atypical symptoms or extraesophageal manifestations, such as chronic cough, laryngitis, asthma, non-cardiac chest pain, postnasal drip, recurrent sinusitis, and dental erosion [1, 6–8].

Extraesophageal reflux (EER) symptoms can occur with or without typical GERD symptoms. According to reports, typical GERD symptoms are significantly associated with atypical symptoms. Approximately 80% of individuals with frequent typical reflux symptoms present at least one atypical symptom [4, 9]. However, despite the increasing recognition of these populations, the efficacy of acid suppression therapy against EER remains controversial [10–27]. Acid suppression with proton pump inhibitors (PPIs) is a mainstream therapy for EER, as well as in typical GERD. However, the response to anti-reflux therapy in patients with extraesophageal symptoms is often less impressive, takes longer to occur, and tends to be more difficult to maintain [28]. In contrast, some studies have reported that high-dose PPIs may be more effective than standard-dose PPIs in treating extraesophageal manifestations of GERD [29–31].

In this randomized, open-label trial, we aimed to assess the acid suppression effects of rabeprazole 20 mg twice daily (high- or double-dose PPI) on the extraesophageal manifestations of GERD compared to those of rabeprazole 20 mg once daily (standard-dose PPI).

## Methods

### Study subjects

This single center, prospective, randomized, open-label, comparative study was conducted in Korea from October 2012 to July 2014. Patients were recruited from the Gastroenterology Center at Seoul National University Bundang Hospital. Inclusion criteria were as follows: 1) patients aged 20 to 80 years; and 2) those who had at least weekly symptoms of typical and/or atypical GERD within a month prior to the start of the study. Typical GERD symptoms were defined as heartburn and regurgitation. Atypical GERD symptoms were defined as chest pain, cough, globus, wheezing, laryngopharyngitis, hoarseness, belching, and dysphagia. Exclusion criteria were as follows: 1) patients with an endoscopic finding of esophageal stricture, esophageal varix, Barrett’s esophagus, peptic ulcer, gastrointestinal bleeding, Zollinger–Ellison syndrome, or malignancy; 2) patients who had undergone a previous gastrointestinal operation, such as an operation to inhibit gastric acid secretion, esophagectomy, or gastrectomy (simple stomach perforation operation was excluded); 3) patients who used histamine-2 receptor antagonists, PPIs, anticholinergic drugs (muscarinic receptor antagonists), gastrin receptor antagonists, protective factor enhancers, gastric mucosal protective agents, or nonsteroidal anti-inflammatory drugs (NSAIDs) within 4 weeks of the screening test; 4) women who were pregnant or lactating; 5) women of childbearing age not using contraception; and 6) patients with significant impairments in the hematologic, renal, cardiac, pulmonary, hematopoietic, or endocrine systems.

### Study protocol

**Fig. 1:**
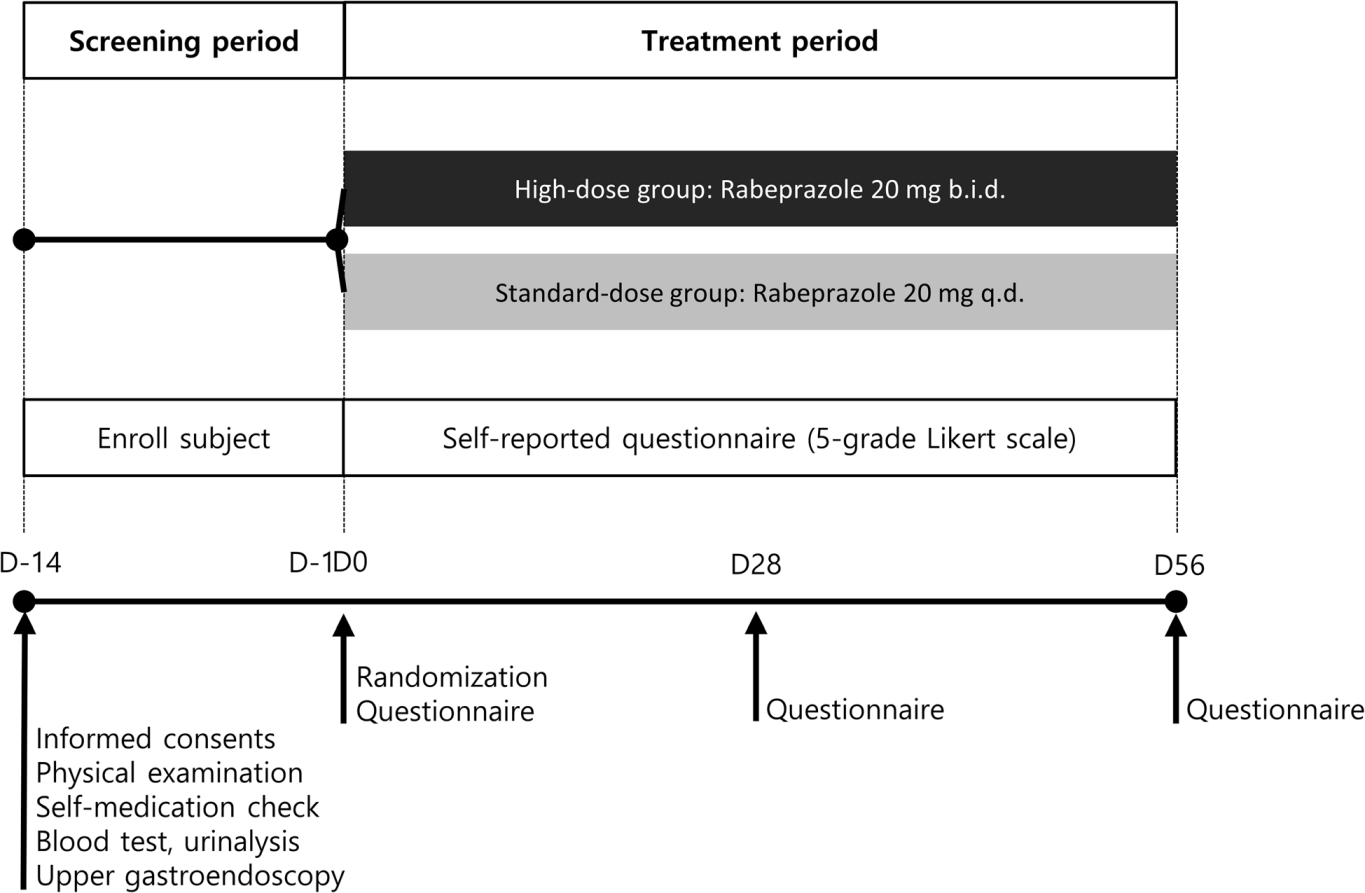
Overview of the study design. *q.d.* Once daily, *b.i.d.* twice daily

Subjects who participated in the clinical study underwent blood tests, urinalysis, and an upper gastroendoscopy as screening tests. Patients eligible for the screening test were sequentially assigned into 2 groups in randomly: 1) standard-dose group received 20 mg rabeprazole once daily before breakfast, and 2) high-dose group received 20 mg rabeprazole twice daily before breakfast and dinner for 8 weeks. The randomization was conducted by the study staff using a 1:1 ratio in 20 blocks sizes of 6. As the duration of first-line treatment generally recommended for GERD is 8 to 12 weeks, and the response to treatment for extraesophageal manifestations of GERD is slower than that for typical symptoms, we determined that a treatment period of at least 8 weeks was necessary. The endoscopic findings were classified according to the modified Los Angeles (LA) classification system of GERD (N, normal; M, minimal change; A–D, grade A–D) [32–35], and a rapid urease test for detecting *Helicobacter pylori* (*H. pylori*) infection was performed. Compliance was determined by the number of remaining tablets per drug type at the follow-up visit. The study design is summarized in Fig. 1.

### Questionnaires

Patients visited the clinical study center and completed the self-administered questionnaire to evaluate the symptoms of GERD at baseline before treatment and at weeks 4 and 8. The questionnaire comprised 10 items including 2 questions on typical GERD symptoms (heartburn and acid regurgitation) and 8 questions on atypical GERD symptoms (chest pain, cough, globus, wheezing, laryngopharyngitis, hoarseness, belching, and dysphagia). A 5-grade Likert scale was used in the questionnaire to assess the intensity of symptoms (0, no symptoms; 1, mild symptoms that are not easily felt; 2, moderate symptoms that do not affect daily life; 3, severe symptoms that hinder daily life or sleep; 4, very severe symptoms that do not allow normal daily life or sleep).

### Efficacy assessments

The efficacy of the treatments was assessed by evaluating the mean of the questionnaire symptom scores. The change in reflux symptoms was classified as very much improved (symptom reduction ≥75%), much improved (≥ 50% but < 75% symptom reduction), minimally improved (≥ 25% but < 50% symptom reduction), or no change (< 25% symptom reduction). The primary endpoint was sufficient improvement of reflux symptoms after 8 weeks of treatment. Sufficient improvement of reflux symptoms was defined as ≥50% reduction (very much or much improved) from the initial questionnaire score. The secondary endpoint was to compare the difference in each score after treatment between the two groups for each symptom.

### Safety assessments

Safety assessments included adverse events (AEs) and adverse drug reactions (ADRs), including any gastrointestinal symptoms and abnormalities in laboratory findings or vital signs. Complaint questionnaires were administered to assess for any harmful or untoward reactions experienced by a patient.

### Sample size and statistical analysis

This study assumed that the therapeutic effect of the standard-dose PPI was 50% and the high-dose PPI was 80%, based on the results of previous studies on the efficacy of PPI in extraesophageal manifestations of GERD [16, 19–26]. On the basis of this assumption of a difference of 30% to yield a statistical power of 0.80 with an α -value of 0.05, the total number of participants required for the study should be approximately 120 (for each group of 60 subjects assuming a 20% drop-out rate), as analyzed by G*Power version 3.1 software (Dusseldorf, Germany). Repeated measures analysis of variance (RM-ANOVA) was used to evaluate differences in efficacy of drug treatments between the two groups. The effective rate was analyzed by a Chi square (χ^2^) or Fisher’s exact test. Inter-group comparisons of the other variables were conducted using the Student’s *t*-test or Mann-Whitney U test for continuous data and the χ^2^ or Fisher’s exact test for categorical data. All statistical analyses were performed using SPSS, version 25.0 (IBM Inc., Chicago, IL, USA).

### Ethics statement

This study was reviewed by the Institutional Review Board of Seoul National University Bundang Hospital (B-1008-110-005). All procedures performed in this study involving human participants were in accordance with the 1964 Declaration of Helsinki and its later amendments or comparable ethical standards. Informed consent was submitted by all subjects when they were enrolled. This study was registered as a standard, randomized clinical trial (ClinicalTrials.gov: NCT04001400).

## Results

### Allocation of patients

**Fig. 2:**
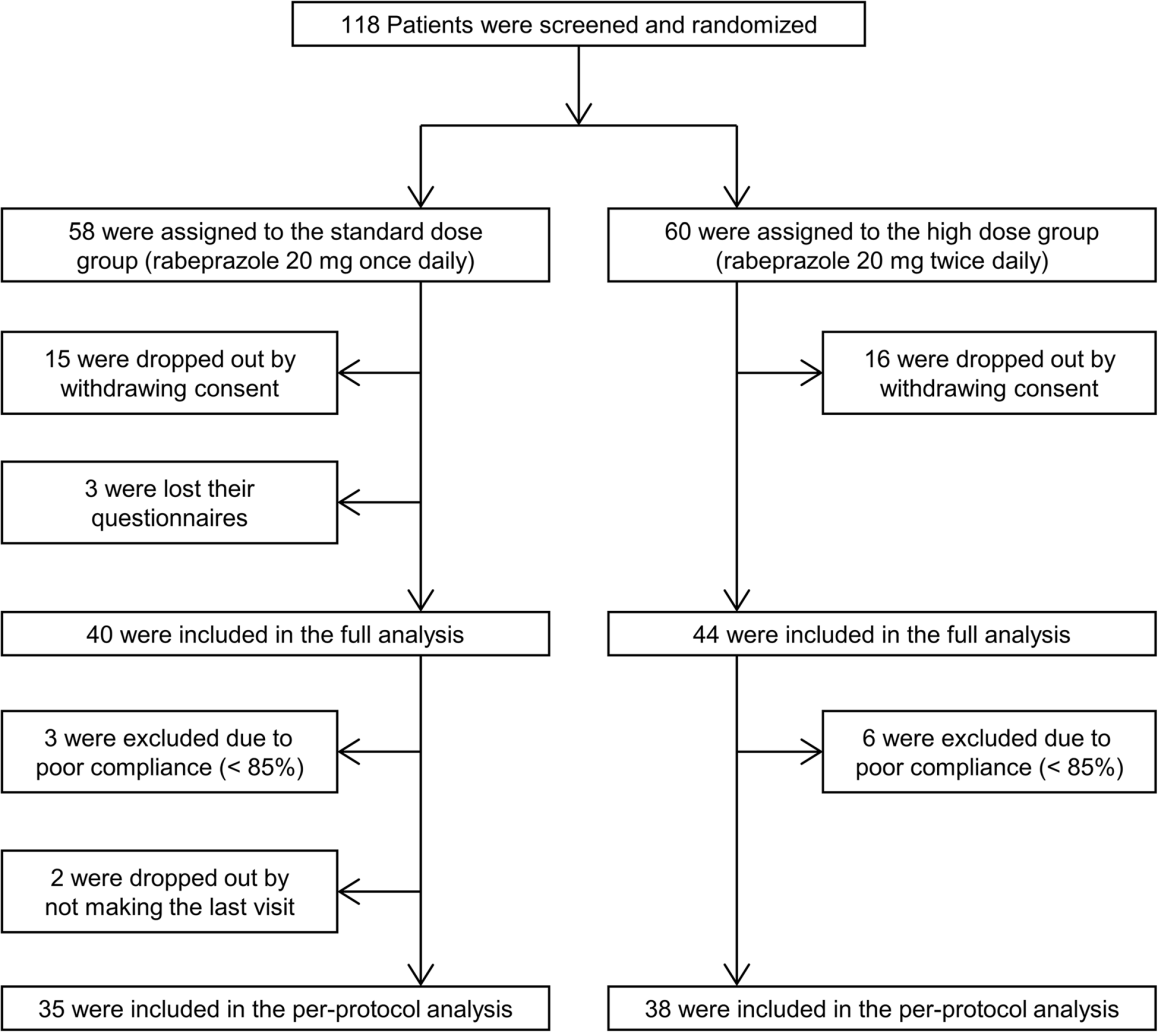
Flow diagram of study patients

A total of 118 patients signed informed consent forms and participated in the screening. All of them met the inclusion criteria and were randomly assigned to the standard-dose group or the high-dose group. However, 31 patients withdrew their consent before the beginning of treatment, and the remaining 87 patients completed the study. Unfortunately, 3 patients lost their questionnaires, and a total of 84 patients were included for the intention-to-treat (ITT) analysis. After 8 weeks of treatment, 9 patients with low drug compliance (< 85%) and 2 patients who did not make the last visit had to be excluded for the per-protocol (PP) analysis. Figure 2 presents the flow of study patients.

### Demographic characteristics

Table 1 shows patients’ demographic and baseline characteristics. There were no differences between the two groups in terms of age, sex, height, weight, body mass index, or smoking status. The other clinical factors, including history of angina, any use of anti-psychotics or antidepressants, LA classification grades, status of *H. pylori* infection, or symptom scores in the questionnaire prior to treatment, were also not significantly different between groups.

**Table 1:** Baseline Characteristics of the Study Participants

Variables	Standard-dose group^a^ (*n* = 40)	High-dose group^b^ (*n* = 44)	*P-value*
Age, years	56.3 ± 12.4	59.2 ± 9.4	0.223
Sex, male:female	12:28 (30.0:70.0)	15:29 (34.1:65.9)	0.693
Height, cm	161.6 ± 6.8	160.0 ± 8.4	0.357
Weight, Kg	60.5 ± 11.9	59.2 ± 11.4	0.602
BMI, Kg/m^2^ (range)	23.0 ± 3.3 (17.3–31.8)	22.9 ± 3.0 (16.2–29.7)	0.889
Smoking status			
Non-smoker	35 (87.5)	41 (93.2)	0.382
Current/ex-smoker	5 (12.5)	3 (6.8)	0.382
History of angina	1 (2.5)	2 (4.5)	0.619
Use of anti-psychotic drug	8 (20.0)	11 (25.0)	0.590
Reflux esophagitis (LA grade)			
Normal	7 (17.5)	7 (15.9)	0.847
Minimal change	24 (60.0)	26 (59.1)	0.933
A	7 (17.5)	8 (18.2)	0.936
B	1 (2.6)	3 (6.8)	0.373
C	1 (2.6)	0 (0.0)	0.323
*H. pylori*			
Positive	7 (17.5)	10 (22.7)	0.557
Previous history of eradication	6 (15.0)	7 (15.9)	0.807
Initial symptom score^c^			
Total symptoms	4.4 ± 2.8	4.9 ± 2.2	0.337
Typical symptoms^d^	2.6 ± 1.1	2.5 ± 1.1	0.815
Atypical symptoms^e^	3.2 ± 2.0	3.3 ± 1.7	0.897

*P*-values were calculated using Student’s *t*-test or χ^2^-test

Values are presented as mean ± standard deviation or *n* (%)

*BMI* body mass index, *LA* Los Angeles, *H. pylori* Helicobacter pylori

^a^Standard-dose group was treated as rabeprazole 20 mg once daily

^b^High-dose group was treated as rabeprazole 20 mg twice daily

^c^Symptom score was the mean value of the score sum of the 5-graded Likert scale questionnaire

^d^Typical symptoms were defined as heartburn and regurgitation

^e^Atypical symptoms were defined as chest pain, cough, globus, wheezing, laryngopharyngitis, hoarseness, belching, and dysphagia

### Primary efficacy endpoint

Table 2 shows the proportion of study subjects who had sufficient improvement (≥ 50% reduction of the symptom scores in the questionnaire) of reflux symptoms after 8 weeks of treatment between the two groups. On the basis of both ITT and PP analyses, the proportion of patients with sufficient improvement of total GERD symptoms was significantly higher in the high-dose group than in the standard-dose group (ITT: 88.6% vs. 67.5%, *P* = 0.018; PP: 92.1% vs. 68.6%, *P* = 0.004). The proportion of patients with sufficient improvement of typical GERD symptoms was also significantly higher in the high-dose group than in the standard-dose group (ITT: 100.0% vs. 82.8%, *P* = 0.018; PP: 100.0% vs. 84.0%, *P* = 0.040). For atypical GERD symptoms, the proportion of patients with sufficient symptom improvement tended to be higher in the high-dose group than in the standard-dose group, which did not reach a statistical significance (ITT: 79.5% vs. 64.5%, *P* = 0.162; PP: 82.4% vs. 63.0%, *P* = 0.087). Subgroup analysis by sex was performed. On the basis of the ITT analysis, in a subgroup of men, the high-dose group had more improvement of atypical GERD symptoms than the standard-dose group (93.3% vs. 55.6%, *P* = 0.047). However, no sex differences were observed on the PP analysis.

**Table 2:** Intention-to-treat (ITT) and per-protocol (PP) analysis of the Efficacy of the Sufficient Improvement^a^ of Reflux symptoms between the Two Groups

	Standard-dose group^b^		High-dose group^c^		*P*-value
	*n/N* (%)	95% CI	*n/N* (%)	95% CI	
ITT analysis					
Total symptoms					
All gender	27/40 (67.5)	60.0–75.0	39/44 (88.6)	83.8–93.4	**0.018**
Male	7/12 (58.3)	43.4–73.2	14/15 (93.3)	86.6–100.0	0.060
Female	20/28 (71.4)	62.7–80.1	25/29 (86.2)	79.7–92.7	0.171
Typical symptoms^d^					
All gender	24/29 (82.8)	75.7–89.9	33/33 (100.0)	100.0–100.0	**0.018**
Male	6/8 (75.0)	58.6–91.4	10/10 (100.0)	100.0–100.0	0.183
Female	18/21 (85.7)	77.9–93.5	23/23 (100.0)	100.0–100.0	0.100
Atypical symptoms^e^					
All gender	20/31 (64.5)	55.8–73.2	31/39 (79.5)	72.9–86.1	0.162
Male	5/9 (55.6)	38.0–73.2	14/15 (93.3)	86.6–100.0	**0.047**
Female	15/22 (68.2)	58.0–78.4	17/24 (70.8)	61.3–80.3	0.845
PP analysis					
Total symptoms					
All gender	24/35 (68.6)	60.6–76.6	35/38 (92.1)	87.7–96.5	**0.011**
Male	7/11 (63.6)	48.4–78.8	13/14 (92.9)	85.8–100.0	0.133
Female	17/24 (70.8)	61.3–80.3	22/24 (91.7)	87.1–98.7	0.137
Typical symptoms^d^					
All gender	21/25 (84.0)	76.5–91.5	29/29 (100.0)	100.0–100.0	**0.040**
Male	6/8 (75.0)	58.6–91.4	10/10 (100.0)	100.0–100.0	0.183
Female	15/17 (88.2)	80.1–96.3	19/19 (100.0)	100.0–100.0	0.216
Atypical symptoms^e^					
All gender	17/27 (63.0)	53.5–72.5	28/34 (82.4)	75.8–89.0	0.087
Male	5/8 (62.5)	44.2–80.8	13/14 (92.9)	85.8–100.0	0.117
Female	12/19 (63.2)	51.8–74.6	15/20 (75.0)	65.1–84.9	0.423

### Secondary efficacy endpoint

Table 3 shows the results of stratified analysis of GERD symptom scores on the questionnaire in time-course changes, on the basis of the PP analysis (The ITT analysis showed the same results and is presented in Supplementary Table S1). Scores of total and typical symptoms, including heartburn and acid regurgitation, significantly improved at 8 weeks with intergroup differences (ITT and PP: all *P* < 0.05). With respect to atypical symptoms, although there was no difference between the two groups, the same tendency was maintained (ITT: *P* = 0.128; PP: *P* = 0.079). There were significant inter-group differences only in cough and globus, among atypical GERD symptoms (ITT: *P* = 0.020 and *P* = 0.027; PP: *P* = 0.044 and *P* < 0.001). At least one of the two groups had no wheezing, laryngopharyngitis, and dysphagia symptoms.

**Table 3:** Time-Course Changes of Reflux Symptom Scores^a^ between the Two Groups

	Standard-dose group^b^ (*n* = 35)				High-dose group^c^ (*n* = 38)				*P*-value
	*n*	Baseline	4 week (△)	8 week (△)	*n*	Baseline	4 week (△)	8 week (△)	
Total symptoms	35	4.46 ± 2.76	2.09 ± 1.96 (−2.37 ± 1.70)	1.83 ± 1.79 (− 2.63 ± 1.78)	38	4.76 ± 2.01	0.92 ± 1.42 (−3.84 ± 1.98)	0.89 ± 1.37 (−3.87 ± 1.85)	**< 0.001**
Typical symptoms^d^	25	2.56 ± 1.12	1.16 ± 1.34 (− 1.40 ± 1.26)	0.80 ± 0.91 (− 1.76 ± 0.83)	29	2.69 ± 1.00	0.28 ± 0.60 (− 2.41 ± 1.24)	0.24 ± 0.44 (− 2.45 ± 1.09)	**0.004**
Heartburn	21	2.14 ± 0.73	0.90 ± 1.04 (− 1.24 ± 0.83)	0.76 ± 0.89 (− 1.38 ± 0.74)	19	2.26 ± 0.56	0.26 ± 0.65 (− 2.00 ± 0.82)	0.21 ± 0.42 (− 2.05 ± 0.71)	**0.004**
Acid regurgitation	11	1.73 ± 0.65	0.36 ± 0.51 (− 1.36 ± 0.51)	0.36 ± 0.51 (− 1.36 ± 0.51)	16	2.19 ± 0.54	0.19 ± 0.40 (− 2.00 ± 0.63)	0.19 ± 0.40 (− 2.00 ± 0.52)	**0.005**
Atypical symptoms^e^	27	3.41 ± 1.93	1.85 ± 1.51 (− 1.56 ± 1.19)	1.56 ± 1.28 (− 1.85 ± 1.35)	34	3.03 ± 1.14	0.79 ± 1.23 (− 2.24 ± 1.28)	0.79 ± 1.27 (− 2.24 ± 1.26)	0.079
Chest pain	7	2.29 ± 0.49	1.14 ± 0.90 (− 1.14 ± 0.90)	0.71 ± 0.76 (− 1.57 ± 0.54)	11	2.27 ± 0.65	0.91 ± 0.94 (− 1.36 ± 0.92)	0.82 ± 1.08 (− 1.45 ± 0.93)	0.692
Cough	6	2.17 ± 0.75	1.50 ± 1.23 (− 0.67 ± 0.82)	1.33 ± 1.03 (− 0.83 ± 0.75)	8	2.25 ± 0.71	0.38 ± 0.74 (− 1.87 ± 1.13)	0.38 ± 0.52 (− 1.87 ± 1.13)	**0.044**
Globus	20	2.30 ± 0.66	1.35 ± 0.75 (− 0.95 ± 0.61)	1.20 ± 0.77 (− 1.10 ± 0.72)	17	2.47 ± 0.51	0.47 ± 0.80 (− 2.00 ± 0.79)	0.53 ± 0.80 (− 1.94 ± 0.75)	**< 0.001**
Wheezing	1	2.00 ± 0.00	1.00 (− 1.00)	1.00 (− 1.00)	0	–	–	–	–
Laryngopharyngitis	0	–	–	–	0	–	–	–	–
Hoarseness	2	2.00 ± 0.00	0.50 ± 0.71 (−1.50 ± 0.71)	0.50 ± 0.71 (− 1.50 ± 0.71)	4	3.00 ± 0.00	1.50 ± 1.29 (− 1.50 ± 1.29)	1.50 ± 1.29 (− 1.50 ± 1.29)	1.000
Belching	5	2.20 ± 0.45	0.80 ± 1.01 (− 1.40 ± 0.89)	0.60 ± 0.89 (− 1.60 ± 0.55)	3	1.33 ± 0.58	0.00 ± 0.00 (− 1.33 ± 0.58)	0.00 ± 0.00 (− 1.33 ± 0.58)	0.822
Dysphagia	0	–	–	–	1	2.00 ± 0.00	0.00 (− 2.00)	0.00 (− 2.00)	–

*P-*values were calculated using repeated measure ANOVA

Values are presented as mean ± standard deviation

^a^Symptom score was the mean value of the score sum of the 5-graded Likert scale questionnaire

^b^Standard-dose group was treated as rabeprazole 20 mg once daily

^c^High-dose group was treated as rabeprazole 20 mg twice daily

^d^Typical symptoms were defined as heartburn and regurgitation

^e^Atypical symptoms were defined as chest pain, cough, globus, wheezing, laryngopharyngitis, hoarseness, belching, and dysphagia

Bold style indicates statistical significance

### Safety

During the study period, AEs were not reported at all in both groups (data not shown).

## Discussion

This study demonstrated that rabeprazole 20 mg twice daily (high-dose) for up to 8 weeks was more effective than rabeprazole 20 mg once daily (standard-dose) in improving overall symptoms in patients with GERD. When sub-classified into typical and atypical GERD symptoms, typical symptoms of heartburn and acid regurgitation were significantly improved in the high-dose rabeprazole group compared to the standard-dose group. However, with regard to atypical symptoms, only cough and globus sensation were improved in the high-dose group compared to the standard-dose group. The results were the same when analyzed in patients with erosive esophagitis, except for 14 patients with non-erosive GERD (data not shown).

There was no conclusive evidence on the efficacy of PPI treatment for the extraesophageal manifestation of GERD. Several meta-analyses reported conflicting results [10–13]. One meta-analysis showed significant effects of PPI therapy on non-cardiac chest pain compared to placebo (Odd ratio (OR) 0.54, 95% confidence interval (CI) 0.41–0.71) [10]. However, three other meta-analyses reported that there were no statistically significant differences, although PPI therapy tended to have clinical benefit over placebo in GERD-related chronic cough and laryngeal symptoms (OR 0.46, 95% CI 0.19–1.15; risk ratio (RR) 1.28, 95% CI 0.94–1.74; RR 1.18, 0.81–1.74; respectively; RR more than 1 favors PPI) [11–13]. The studies cited in these meta-analyses were placebo-controlled trials of PPIs used at either high- or standard-dose [14–26]. Studies have also been conducted to compare the efficacy of high-dose PPI and standard-dose PPI therapies for extraesophageal symptoms [29, 30]. Park et al. reported that lansoprazole 30 mg twice daily (15/30, 50%) appeared to be more effective than esomeprazole 40 mg once daily (7/25, 28%) for 2 months in achieving symptomatic relief in laryngopharyngeal reflux [29]. Kiljander et al. demonstrated that only esomeprazole 40 mg twice daily, not once daily, significantly improved pulmonary function in asthma over the entire 26-week period in patients with concomitant symptoms of GERD [30]. Our findings provide additional evidence, indicating not only that PPI treatment is effective against extraesophageal manifestation of GERD, but also that high-dose PPI is more beneficial than standard-dose PPI.

It is well known that typical symptoms of heartburn and acid regurgitation are significantly associated with atypical symptoms such as non-cardiac chest pain, dysphagia, dyspepsia, asthma, hoarseness, and globus sensation [4, 9]. Locke et al. reported that the prevalence rate of any atypical symptom in patients with typical GERD symptoms at least weekly was 79.9% (242/303) [4]. Dore at al. reported that the occurrence of atypical symptoms was approximately equal to that of typical symptoms in patients with erosive GERD (119/166, 71.7%) and with non-erosive GERD (79/100, 79.0%) [9]. The subjects of our study are same as those of previous studies. The proportion of subjects presenting any atypical symptom among those with typical symptom was 77.4% (48/62, Supplementary Table S2). Furthermore, our results are also consistent with previous reports showing that PPIs are less effective against atypical symptoms than against typical symptoms, regardless of the PPI dose group (Typical symptoms vs. atypical symptoms; ITT: 57/62, 91.9% vs. 51/70, 72.9%, *P* = 0.005; PP: 50/54, 92.6% vs. 45/61, 73.8%, *P* = 0.008; Supplementary Table S3).

There are various explanations for why the treatment of atypical symptoms is more difficult than typical symptoms. As one possibility, human studies suggest that nocturnal gastroesophageal reflux is more common in this group of patients. Thus, a once daily regimen may be insufficient for improving these symptoms, and these studies are the reason for nocturnal dosing of PPIs [36, 37]. It is also possible that the diagnosis of GERD may not be valid. Experts recommend that obtaining an accurate history and documenting the presence of a baseline reflux with appropriate investigations (i.e., endoscopy and pH monitoring) are important for patients presenting extraesophageal symptoms with an incomplete PPI response [8, 38]. Nonetheless, therapeutic trials using high-dose PPI may be helpful. Charbel et al. revealed that the likelihood of normal pH values was 11 times higher for patients on twice daily PPIs than those on once daily PPIs by pH monitoring in either typical or extraesophageal GERD patients (OR 11.4, 95% CI 4.3–30.1) [39]. Rabeprazole, the PPI chosen in our study, can be activated at a higher pH than other PPIs owing to a high pKa of approximately 5.0. This possibly results in faster onset of action. Owing to its non-enzymatic pathway of metabolism, rabeprazole is also less influenced by genetic polymorphisms of CYP2C19 that promote metabolism of other PPIs [40]. These pharmacodynamic properties support the therapeutic effect observed in our study.

This study had some limitations. First, the atypical symptoms included in the questionnaire were heterogenous. The mixture of symptoms such as non-cardiac chest pain and chronic cough made it difficult to interpret the therapeutic effect. Second, our 5-grade Likert scale questionnaire was not validated for GERD. However, we chose an easily available method for patients with clinical situation. This study was not conducted with subjects recruited through advertisement. Most of the study participants were patients who were transferred to the tertiary hospital because their GERD symptoms did not improve despite treatment at the primary clinics. So, it was not easy to enroll these patients in the study. Since these patients did not visit our hospital for the purpose of participating in the research, to lead participation, the questionnaire should be simple and understandable rather than complicated and laborious. Therefore, we used the subjective questionnaire, which could result in bias. Furthermore, EER was a disease that complains of subjective symptoms, and did not have specific criteria. We determined to adapt the questionnaire considering these characteristics of the disease and the clinical situation of the participants. And, we planned it as an open-label trial rather than a double-blind considering compliance. As mentioned above, patients visiting for the purpose of treatment were often unwilling to participate in the research, so, a considerable number of subjects withdrew their participation at the beginning of medication. Third, we did not confirm the validity of the diagnosis of GERD. Although upper gastroendoscopy was performed to reveal esophageal mucosal injury, other investigations such as 24-h pH or pH-impedance monitoring, laryngoscopy, or manometry were not used, especially in subjects with non-erosive GERD. Thus, we could not make sure that the subject without response to PPI treatment had no pathologic gastric acid reflux into the esophagus and laryngopharynx, or insufficient acid suppression by PPI. Fourth, we did not know the complete remission of esophageal mucosal healing, because we could not perform upper gastroendoscopy after treatment. We focused on the quality of life as an endpoint in GERD treatment. Fifth, the placebo effect of twice daily regimen of PPI for once daily regimen cannot be overlooked. This disorder, characterized by subjective symptoms rather than objective findings, is prone to a placebo response [19]. We did not administer any placebo at nighttime in the standard-dose group corresponding to the evening dose in the high-dose group. Sixth, we did not exclude patients with psychological history. The importance of central mechanisms involved in functional gastrointestinal disorders and gastroenterologists’ understanding of the relationship of symptoms to anxiety or depression has increased. Moreover, it is known that the rate of psychiatric illness in patients with these gastrointestinal disorders is high [41]. In real clinical situation, not a few patients with GERD who respond incompletely to standard-dose PPI seem to be treated with anti-psychotics. Therefore, excluding participants with psychological history or taking anti-psychotics may rather induce selection bias or weaken the clinical significance for real practice.

## Conclusions

This randomized, open-label study demonstrated that treatment with high-dose rabeprazole (20 mg twice daily) was more effective than standard-dose rabeprazole (20 mg once daily) for typical GERD symptoms, and also suggested that treatment with high-dose rabeprazole tended to be more effective than standard-dose rabeprazole for atypical GERD symptoms. Further investigations are warranted to support these findings.

## Supplementary information

**Additional file 1. Supplementary Table S1.** Time-Course Changes of Reflux Symptom Scores between the Two Groups. **Supplementary Table S2.** Frequency Distribution of Atypical Symptom among GERD Patients with Typical Symptom. **Supplementary Table S3.** Intention-to-treat (ITT) and per-protocol (PP) analysis of the Rate of the Sufficient Improvement according to GERD symptoms.

## Data Availability

The datasets used and/or analysed during the current study are available from the corresponding author on reasonable request.

## Abbreviations

GERD: Gastroesophageal reflux disease
EER: Extraesophageal reflux
PPI: Proton pump inhibitor
NSAIDs: Nonsteroidal anti-inflammatory drugs
LA: Los Angeles
AEs: Adverse events
ADRs: Adverse drug reactions
*H. pylori*: *Helicobacter pylori*
OR: Odd ratio
CI: Confidence interval
RR: Risk ratio

## References

[1] Vakil N, van Zanten SV, Kahrilas P, Dent J (2006). Jones R; global consensus group. The Montreal definition and classification of gastroesophageal reflux disease: a global evidence-based consensus. Am J Gastroenterol.

[2] Hunt R, Armstrong D, Katelaris P, Afihene M, Bane A, Bhatia S (2017). World gastroenterology organisation global guidelines: GERD global perspective on Gastroesophageal reflux disease. J Clin Gastroenterol.

[3] El-Serag HB, Sweet S, Winchester CC, Dent J (2014). Update on the epidemiology of gastro-oesophageal reflux disease: a systematic review. Gut..

[4] Locke GR, Talley NJ, Fett SL, Zinsmeister AR, Melton LJ (1997). Prevalence and clinical spectrum of gastroesophageal reflux: a population-based study in Olmsted County, Minnesota. Gastroenterology..

[5] Cho YS, Choi MG, Jeong JJ, Chung WC, Lee IS, Kim SW (2005). Prevalence and clinical spectrum of gastroesophageal reflux: a population-based study in Asan-si, Korea. Am J Gastroenterol.

[6] Vaezi MF, Katzka D, Zerbib F (2018). Extraesophageal symptoms and diseases attributed to GERD: where is the pendulum swinging now?. Clin Gastroenterol Hepatol.

[7] Saritas Yuksel E, Vaezi MF (2012). New developments in extraesophageal reflux disease. Gastroenterol Hepatol.

[8] Zerbib F, Dulery C (2017). Facts and fantasies on Extraesophageal reflux: a gastroenterologist perspective. J Clin Gastroenterol.

[9] Dore MP, Pedroni A, Pes GM, Maragkoudakis E, Tadeu V, Pirina P (2007). Effect of antisecretory therapy on atypical symptoms in gastroesophageal reflux disease. Dig Dis Sci.

[10] Cremonini F, Wise J, Moayyedi P, Talley NJ (2005). Diagnostic and therapeutic use of proton pump inhibitors in non-cardiac chest pain: a metaanalysis. Am J Gastroenterol.

[11] Chang AB, Lasserson TJ, Gaffney J, Connor FL, Garske LA (2011). Gastro-oesophageal reflux treatment for prolonged non-specific cough in children and adults. Cochrane Database Syst Rev.

[12] Qadeer MA, Phillips CO, Lopez AR, Steward DL, Noordzij JP, Wo JM (2006). Proton pump inhibitor therapy for suspected GERD-related chronic laryngitis: a meta-analysis of randomized controlled trials. Am J Gastroenterol.

[13] Gatta L, Vaira D, Sorrenti G, Zucchini S, Sama C, Vakil N (2007). Meta-analysis: the efficacy of proton pump inhibitors for laryngeal symptoms attributed to gastro-oesophageal reflux disease. Aliment Pharmacol Ther.

[14] TO K, Salomaa ER, Hietanen EK, Terho EO (2000). Chronic cough and gastro-oesophageal reflux: a double-blind placebo-controlled study with omeprazole. Eur Respir J.

[15] Langevin S, Hanh N (2001). GERD-induced ENT symptoms: A prospective placebo controlled study with omeprazole 40 mg a day. Gastroenterology.

[16] Wo JM, Koopman J, Harrell SP, Parker K, Winstead W, Lentsch E (2006). Double-blind, placebo-controlled trial with single-dose pantoprazole for laryngopharyngeal reflux. Am J Gastroenterol.

[17] Pawar S, Lim HJ, Gill M, Smith TL, Merati A, Toohill RJ, Loehrl TA (2007). Treatment of postnasal drip with proton pump inhibitors: a prospective, randomized, placebo-controlled study. Am J Rhinol.

[18] El-Serag HB, Lee P, Buchner A, Inadomi JM, Gavin M, McCarthy DM (2001). Lansoprazole treatment of patients with chronic idiopathic laryngitis: a placebo-controlled trial. Am J Gastroenterol.

[19] Noordzij JP, Khidr A, Evans BA, Desper E, Mittal RK, Reibel JF, Levine PA (2001). Evaluation of omeprazole in the treatment of reflux laryngitis: a prospective, placebo-controlled, randomized, double-blind study. Laryngoscope..

[20] Ours TM, Kavuru MS, Schilz RJ, Richter JE (1999). A prospective evaluation of esophageal testing and a double-blind, randomized study of omeprazole in a diagnostic and therapeutic algorithm for chronic cough. Am J Gastroenterol.

[21] Lam PK, Ng ML, Cheung TK, Wong BY, Tan VP, Fong DY (2010). Rabeprazole is effective in treating laryngopharyngeal reflux in a randomized placebo-controlled trial. Clin Gastroenterol Hepatol.

[22] Vaezi MF, Richter JE, Stasney CR, Spiegel JR, Iannuzzi RA, Crawley JA (2006). Treatment of chronic posterior laryngitis with esomeprazole. Laryngoscope..

[23] Steward DL, Wilson KM, Kelly DH, Patil MS, Schwartzbauer HR, Long JD, Welge JA (2004). Proton pump inhibitor therapy for chronic laryngo-pharyngitis: a randomized placebo-control trial. Otolaryngol Head Neck Surg.

[24] Havas T, Huang S, Levy M, Abi-Hanna D (1999). Posterior pharyngolaryngitis: double blind randomized placebo-controlled trial of proton pump inhibitor therapy. Aust J Otolaryng.

[25] Eherer AJ, Habermann W, Hammer HF, Kiesler K, Friedrich G, Krejs GJ (2003). Effect of pantoprazole on the course of reflux-associated laryngitis: a placebo-controlled double-blind crossover study. Scand J Gastroenterol.

[26] Shaheen NJ, Crockett SD, Bright SD, Madanick RD, Buckmire R, Couch M (2011). Randomised clinical trial: high-dose acid suppression for chronic cough - a double-blind, placebo-controlled study. Aliment Pharmacol Ther.

[27] Kahrilas PJ, Howden CW, Hughes N, Molloy-Bland M (2013). Response of chronic cough to acid-suppressive therapy in patients with gastroesophageal reflux disease. Chest..

[28] Fennerty MB (1999). Extraesophageal gastroesophageal reflux disease. Presentations and approach to treatment. Gastroenterol Clin N Am.

[29] Park W, Hicks DM, Khandwala F, Richter JE, Abelson TI, Milstein C, Vaezi MF (2005). Laryngopharyngeal reflux: prospective cohort study evaluating optimal dose of proton-pump inhibitor therapy and pretherapy predictors of response. Laryngoscope..

[30] TO K, Junghard O, Beckman O, Lind T (2010). Effect of esomeprazole 40 mg once or twice daily on asthma: a randomized, placebo-controlled study. Am J Respir Crit Care Med.

[31] Hershcovici T, Achem SR, Jha LK, Fass R (2012). Systematic review: the treatment of noncardiac chest pain. Aliment Pharmacol Ther.

[32] Armstrong D, Bennett JR, Blum AL, Dent J, De Dombal FT, Galmiche JP (1996). The endoscopic assessment of esophagitis: a progress report on observer agreement. Gastroenterology..

[33] Lundell LR, Dent J, Bennett JR, Blum AL, Armstrong D, Galmiche JP (1999). Endoscopic assessment of oesophagitis: clinical and functional correlates and further validation of the Los Angeles classification. Gut..

[34] Hoshihara Y (2004). Endoscopic findings of GERD. Nippon Rinsho.

[35] Hongo M (2006). Minimal changes in reflux esophagitis: red ones and white ones. J Gastroenterol.

[36] Kamel PL, Hanson D, Kahrilas PJ (1994). Omeprazole for the treatment of posterior laryngitis. Am J Med.

[37] Vaezi MF (2005). Atypical manifestations of gastroesophageal reflux disease. MedGenMed..

[38] Kahrilas PJ, Boeckxstaens G, Smout AJ (2013). Management of the patient with incomplete response to PPI therapy. Best Pract Res Clin Gastroenterol.

[39] Charbel S, Khandwala F, Vaezi MF (2005). The role of esophageal pH monitoring in symptomatic patients on PPI therapy. Am J Gastroenterol.

[40] Dadabhai A, Friedenberg FK (2009). Rabeprazole: a pharmacologic and clinical review for acid-related disorders. Expert Opin Drug Saf.

[41] Clouse RE, Lustman PJ (2005). Use of psychopharmacological agents for functional gastrointestinal disorders. Gut..

