# Supplementary material for: Efficacy of a high-dose proton pump inhibitor in patients with gastroesophageal reflux disease: a single center, randomized, open-label trial
Source: BMC Gastroenterol. 2020 Aug 18;20:275. doi: 10.1186/s12876-020-01410-z (PMC7433117; doi:10.1186/s12876-020-01410-z)
Supplement: Supplementary file 1 — Additional file 1. Supplementary Table S1. Time-Course Changes of Reflux Symptom Scores between the Two Groups. Supplementary Table S2. Frequency Distribution of Atypical Symptom among GERD Patients with Typical Symptom. Supplementary Table S3. Intention-to-treat (ITT) and per-protocol (PP) analysis of the Rate of the Sufficient Improvement according to GERD symptoms. [file 12876_2020_1410_MOESM1_ESM.docx]

| **Supplementary Table S1.** Time-Course Changes of Reflux Symptom Scores^a^ between the Two Groups | | | | | | | | | | | | | | |
| --- | --- | --- | --- | --- | --- | --- | --- | --- | --- | --- | --- | --- | --- | --- |
|  | Standard-dose group^b^ (*n* = 40) | | | |  | High-dose group^c^ (*n* = 44) | | | | | | | *P*-value | |
|  | *n* | Baseline | 4 week (△) | 8 week (△) |  | *n* | Baseline | | 4 week (△) | | | 8 week (△) |  |  |
| Total symptoms | 40 | 4.43 ± 2.84 | 2.05 ± 1.88  (-2.38 ± 1.78) | 1.80 ± 1.73  (-2.62 ± 1.86) |  | 44 | 4.95 ± 2.17 | | 1.27 ± 1.98  (-3.68 ± 2.06) | | | 1.07 ± 1.44  (-3.89 ± 1.98) | **0.002** | |
| Typical symptoms^d^ | 29 | 2.55 ± 1.12 | 1.21 ± 1.26  (-1.34 ± 1.20) | 0.86 ± 0.88  (-1.69 ± 0.85) |  | 35 | 2.49 ± 1.12 | | 0.31 ± 0.63  (-2.30 ± 1.26) | | | 0.26 ± 0.44  (-2.36 ± 1.08) | **0.015** | |
| Heartburn | 24 | 2.08 ± 0.72 | 0.88 ± 0.99  (-1.21 ± 0.83) | 0.75 ± 0.85  (-1.33 ± 0.76) |  | 23 | 2.26 ± 0.54 | | 0.35 ± 0.71  (-1.91 ± 0.90) | | | 0.26 ± 0.45  (-2.00 ± 0.74) | **0.004** | |
| Acid regurgitation | 14 | 1.71 ± 0.61 | 0.57 ± 0.65  (-1.14 ± 0.66) | 0.50 ± 0.65  (-1.21 ± 0.58) |  | 16 | 2.19 ± 0.54 | | 0.19 ± 0.40  (-2.00 ± 0.63) | | | 0.19 ± 0.40  (-2.00 ± 0.52) | **<0.001** | |
| Atypical symptoms^e^ | 32 | 3.22 ± 1.99 | 1.66 ± 1.49  (-1.56 ± 1.24) | 1.41 ± 1.27  (-1.81 ± 1.38) |  | 40 | 3.28 ± 1.66 | | 1.13 ± 1.83  (-2.21 ± 1.38) | | | 0.95 ± 1.36  (-2.38 ± 1.58) | 0.128 | |
| Chest pain | 7 | 2.29 ± 0.49 | 1.14 ± 0.90  (-1.14 ± 0.90) | 0.71 ± 0.76  (-1.57 ± 0.54) |  | 12 | 2.33 ± 0.65 | | 1.08 ± 1.08  (-1.25 ± 0.97) | | | 0.75 ± 1.06  (-1.58 ± 0.99) | 0.965 | |
| Cough | 8 | 2.13 ± 0.84 | 1.50 ± 1.07  (-0.63 ± 0.74) | 1.38 ± 0.92  (-0.75 ± 0.71) |  | 11 | 2.27 ± 0.65 | | 0.64 ± 1.03  (-1.64 ± 1.12) | | | 0.36 ± 0.51  (-1.91 ± 1.04) | **0.020** | |
| Globus | 21 | 2.33 ± 0.66 | 1.29 ± 0.78  (-1.05 ± 0.74) | 1.14 ± 0.79  (-1.19 ± 0.81) |  | 22 | 2.50 ± 0.51 | | 0.77 ± 1.02  (-1.73 ± 1.03) | | | 0.77 ± 0.97  (-1.73 ± 0.99) | **0.027** | |
| Wheezing | 1 | 2.00 ± 0.00 | 1.00  (-1.00) | 1.00  (-1.00) |  | 0 | - | | - | | | - | - | |
| Laryngopharyngitis | 0 | - | -  - | -  - |  | 1 | 2.00 ± 0.00 | | 1.00  (-1.00) | | | 1.00  (-1.00) | - | |
| Hoarseness | 2 | 2.00 ± 0.00 | 0.50 ± 0.71  (-1.50 ± 0.71) | 0.50 ± 0.71  (-1.50 ± 0.71) |  | 5 | 3.00 ± 0.00 | 1.60 ± 1.14  (-1.40 ± 1.14) | | 1.60 ± 1.14  (-1.40 ± 1.14) | | | | 0.948 |
| Belching | 7 | 2.14 ± 0.38 | 0.57 ± 0.98  (-1.57 ± 0.79) | 0.43 ± 0.79  (-1.71 ± 0.49) |  | 3 | 1.33 ± 0.58 | 0.00 ± 0.00  (-1.33 ± 0.58) | | | 0.00 ± 0.00  (-1.33 ± 0.58) | | | 0.553 |
| Dysphagia | 0 | - | -  - | -  - |  | 1 | 2.00 ± 0.00 | 0.00  (-2.00) | | | 0.00  (-2.00) | | | - |
| *P-*values were calculated using repeated measure ANOVA.  Values are presented as mean ± standard deviation.  ^a^Symptom score was the mean value of the score sum of the 5-graded Likert scale questionnaire.  ^b^Standard-dose group was treated as rabeprazole 20 mg once daily.  ^c^High-dose group was treated as rabeprazole 20 mg twice daily.  ^d^Typical symptoms were defined as heartburn and regurgitation.  ^e^Atypical symptoms were defined as chest pain, cough, globus, wheezing, laryngopharyngitis, hoarseness, belching, and dysphagia.  Bold style indicates statistical significance. | | | | | | | | | | | | | | |

| **Supplementary Table S2.** Frequency Distribution of Atypical Symptom among GERD Patients with Typical Symptom^a^ | | | | |
| --- | --- | --- | --- | --- |
| Atypical symptom | Typical GERD patients | | | *P*-value^b^ |
|  | Total  (*n* = 62) | Erosive GERD  (*n* = 55) | Non-erosive GERD  (*n* = 7) |  |
| Chest pain | 14 (22.6%) | 14 (25.5%) | 0 (0.0%) | 0.334 |
| Cough | 13 (21.0%) | 9 (16.4%) | 4 (57.1%) | **0.030** |
| Globus | 25 (40.3%) | 19 (34.5%) | 6 (85.7%) | **0.014** |
| Wheezing | 1 (1.6%) | 1 (1.8%) | 0 (0.0%) | 1.000 |
| Laryngopharyngitis | 0 (0.0%) | 0 (0.0%) | 0 (0.0%) | - |
| Hoarseness | 6 (9.7%) | 5 (9.1%) | 1 (14.3%) | 1.000 |
| Belching | 7 (11.3%) | 6 (10.9%) | 1 (14.3%) | 1.000 |
| Dysphagia | 1 (1.6%) | 1 (1.8%) | 0 (0.0%) | 1.000 |
| Any atypical symptom | 48 (77.4%) | 42 (76.4%) | 6 (85.7%) | 1.000 |
| *P*-values were calculated using χ^2^-test.  GERD, gastroesophageal reflux disease.  ^a^Typical symptoms were defined as heartburn and regurgitation.  ^b^Erosive GERD *vs*. non-erosive GERD.  Bold style indicates statistical significance. | | | | |

| **Supplementary Table S3.** Intention-to-treat (ITT) and per-protocol (PP) analysis of the Rate of the Sufficient Improvement^a^ according to GERD symptoms | | | | | | | | | |
| --- | --- | --- | --- | --- | --- | --- | --- | --- | --- |
|  | Total symptoms | |  | Typical symptoms^b^ | |  | Atypical symptoms^c^ | | *P*-value^d^ |
|  | *n*/*N* (%) | 95% CI |  | *n*/*N* (%) | 95% CI |  | *n*/*N* (%) | 95% CI |  |
| ITT analysis | | | | | | | | | |
| All gender | 66/84 (78.6) | 74.1–83.1 |  | 57/62 (91.9) | 88.4–95.4 |  | 51/70 (72.9) | 67.5–78.3 | **0.005** |
| Male | 21/27 (77.8) | 69.6–86.0 |  | 16/18 (88.9) | 81.3–96.5 |  | 19/24 (79.2) | 70.7–87.7 | 0.679 |
| Female | 45/57 (78.9) | 73.5–84.3 |  | 41/44 (93.2) | 89.4–97.0 |  | 32/46 (69.6) | 62.7–76.5 | **0.004** |
| PP analysis | | | | | | | | | |
| All gender | 59/73 (80.8) | 76.2–85.4 |  | 50/54 (92.6) | 89.0–96.2 |  | 45/61 (73.8) | 68.1–79.5 | **0.008** |
| Male | 20/25 (80.0) | 71.8–88.2 |  | 16/18 (88.9) | 81.3–96.5 |  | 18/22 (81.8) | 73.4–90.2 | 0.673 |
| Female | 39/48 (81.3) | 75.6–87.0 |  | 34/36 (94.4) | 90.5–98.3 |  | 27/39 (69.2) | 61.7–76.7 | **0.005** |
| *P*-values were calculated using χ^2^-test.  CI, confidence interval.  ^a^Sufficient improvement of reflux symptom was defined as ≥ 50 % reduction from the initial questionnaire score.  ^b^Typical symptoms were defined as heartburn and regurgitation.  ^c^Atypical symptoms were defined as chest pain, cough, globus, wheezing, laryngopharyngitis, hoarseness, belching, and dysphagia.  ^d^Typical symptoms *vs*. atypical symptoms.  Bold style indicates statistical significance. | | | | | | | | | |
